# Mitogenomic evaluation of the historical biogeography of cichlids toward reliable dating of teleostean divergences

**DOI:** 10.1186/1471-2148-8-215

**Published:** 2008-07-23

**Authors:** Yoichiro Azuma, Yoshinori Kumazawa, Masaki Miya, Kohji Mabuchi, Mutsumi Nishida

**Affiliations:** 1 Ocean Research Institute, The University of Tokyo, 1-15-1 Minamidai, Nakano-ku, Tokyo 164-8639, Japan; 2 Division of Material Science and Biological Science, Graduate School of Science, Nagoya University, Furo-cho, Chikusa-ku, Nagoya 464-8602, Japan; 3 Department of Information and Biological Sciences, Graduate School of Natural Sciences, Nagoya City University, 1 Yamanohata, Mizuho-cho, Mizuho-ku, Nagoya 467-8501, Japan; 4 Department of Zoology, Natural History Museum and Institute, Chiba, 955-2 Aoba-cho, Chuo-ku, Chiba 260-8682, Japan

## Abstract

**Background:**

Recent advances in DNA sequencing and computation offer the opportunity for reliable estimates of divergence times between organisms based on molecular data. Bayesian estimations of divergence times that do not assume the molecular clock use time constraints at multiple nodes, usually based on the fossil records, as major boundary conditions. However, the fossil records of bony fishes may not adequately provide effective time constraints at multiple nodes. We explored an alternative source of time constraints in teleostean phylogeny by evaluating a biogeographic hypothesis concerning freshwater fishes from the family Cichlidae (Perciformes: Labroidei).

**Results:**

We added new mitogenomic sequence data from six cichlid species and conducted phylogenetic analyses using a large mitogenomic data set. We found a reciprocal monophyly of African and Neotropical cichlids and their sister group relationship to some Malagasy taxa (Ptychochrominae *sensu *Sparks and Smith). All of these taxa clustered with a Malagasy + Indo/Sri Lankan clade (Etroplinae *sensu *Sparks and Smith). The results of the phylogenetic analyses and divergence time estimations between continental cichlid clades were much more congruent with Gondwanaland origin and Cretaceous vicariant divergences than with Cenozoic transmarine dispersal between major continents.

**Conclusion:**

We propose to add the biogeographic assumption of cichlid divergences by continental fragmentation as effective time constraints in dating teleostean divergence times. We conducted divergence time estimations among teleosts by incorporating these additional time constraints and achieved a considerable reduction in credibility intervals in the estimated divergence times.

## Background

Recent technical advances in the molecular estimation of divergence times have provided molecular evolutionists with promising tools to introduce reliable time scales to molecular phylogenetic trees [1]. One of the most significant advances common to these new methods is the departure from the molecular clock assumption, which in many cases does not strictly hold. Another advance is the use of time constraints at multiple nodes, rather than the assignment of a discrete time value to a particular node, for rate calibration. This is useful because of the various uncertainties in divergence time estimations based on fossil evidence. In general, the occurrence of the earliest fossil assignable to a particular branch can define the lower boundary of divergence time for the node at which this branch departed from its sister branch [2]. However, when the corresponding fossil data are inadequate or sparse, the lower time boundary based on such data could considerably postdate the true divergence time, potentially leading to inaccurate or imprecise dating results [2,3].

In general, fossils of bony fishes are not considered well preserved. Of the 425 teleostean families, 181 families do not have a fossil record. Of the remaining 244 that have fossil records, 58 have only otoliths [4]. Thus, lower boundary values of divergence times based on teleostean fossil evidence could underestimate the true values [5-7]. Therefore, alternative methods that may provide effective time constraints in dating teleostean divergences should be explored, e.g., methods based on reasonable biogeographic assumptions. Because freshwater fishes do not disperse easily through saltwater, their evolution may be tightly linked to the geological history of the landmasses on which they evolved [8,9]. Thus, evaluating the potential correlation of continental drift and lineage divergences in each of the freshwater fish groups that have multicontinental distributions is important [10].

Cichlids (order Perciformes: family Cichlidae) are freshwater fishes that are mainly distributed in landmasses of Gondwanaland origin (Africa, South and Central America, Madagascar, and Indo/Sri Lanka) [11]. They have experienced an explosive radiation in the Great Lakes of East Africa, and they constitute one of the best-known model organisms for evolutionary biology [12]. Phylogenetic studies based on morphological and molecular evidence have consistently recognized the monophyletic origin of the family, basal divergences of Malagasy and Indo/Sri Lankan taxa, and the sister-group relationship of African and South American clades [13-16]. These patterns of divergence among continental cichlid groups are entirely consistent with the geological history of continental drift, the proposed Gondwanan origin of Cichlidae, and subsequent vicariant divergences [5,6,13-18]. However, only a few molecular studies [7,19] have attempted to evaluate this hypothesis by dating cichlid divergences; their different approaches led to opposite conclusions. Genner et al. [7] supported vicariant cichlid divergences during Cretaceous times (vicariant hypothesis), whereas Vences et al. [19] suggested a Cenozoic transmarine dispersal (dispersal hypothesis). The latter conclusion is more consistent with the Eocene occurrence of the oldest cichlid fossils [20].

We used molecular data obtained from complete mitochondrial DNA (mtDNA) sequences to investigate these hypotheses. Among the 54 fish taxa that we sampled, we newly determined the sequence data for six cichlid species. The two alternate hypotheses for cichlids, vicariant and dispersal ones, were evaluated by estimating the divergence times of the taxa using Bayesian analyses that incorporated extensive fossil-based time constraints for various divergences. Despite the relative paucity of fish fossil records, this set of time constraints allowed us to estimate cichlid divergence times with high enough resolution to discriminate between the two alternative hypotheses.

## Methods

### Taxonomic sampling

Cichlid samples were obtained from local animal dealers in Japan. We combined these new mitogenomic data with 48 previously published sequences from the DDBJ/EMBL/GenBank nucleotide sequence database. The 10 cichlid taxa that we analyzed (Table 1) cover species from major Gondwana-origin landmasses. In addition, we chose 31 other teleosts, nine basal actinopterygians, and two sarcopterygians. Two sharks were sampled as an outgroup to root the tree. Additional file 1 contains a complete list of the sampled taxa, along with the database accession numbers of their mitogenomic sequences.

**Table 1:** Cichlid taxa analyzed for mtDNAs

Distribution	Name	mtDNA size (bp)	Accession No.	Reference
Africa	*Tylochromis polylepis*	16876*	AP009509	this study
	*Tropheus duboisi*	16598	AP006015	[43]
	*Oreochromis *sp.	16626	AP009126	[43]
	*Neolamprologus brichardi*	16587	AP006014	[43]
South America	*Astronotus ocellatus*	16569	AP009127	[43]
	*Hypselecara temporalis*	16544	AP009506	this study
Madagascar	*Paratilapia polleni*	16543	AP009508	this study
	*Paretroplus maculatus*	16486	AP009504	this study
	*Ptychochromoides katria*	16556	AP009507	this study
Indo/Sri Lanka	*Etroplus maculates*	16457	AP009505	this study

*Nearly complete mtDNA sequences with the control region partially sequenced

### DNA extraction, PCR, and sequencing

Fish samples were excised from live or dead specimens of each species and immediately preserved in 99.5% ethanol. Total genomic DNA was extracted from muscle, liver, and/or fin clips using a DNeasy tissue kit (Qiagen) or a DNAzol Reagent (Invitrogen), following manufacturer protocols. The mtDNA of each species was amplified using a long-PCR technique with LA-Taq (Takara). Seven fish-versatile primers for long PCR (S-LA-16S-L, L2508-16S, L12321-Leu, H12293-Leu, H15149-CYB, H1065-12S, and S-LA-16S-H [21-26]) and the two cichlid-specific primers cichlid-LA-16SH (5'-TTGCGCTACCTTTGCACGGTCAAAATACCG-3') and cichlid-LA-16SL (5'-CGGAGTAATCCAGGTCAGTTTCTATCTATG-3') were used in various combinations to amplify regions covering the entire mtDNA in one or two reactions. The long-PCR products were used as templates for subsequent short PCR.

Over 100 fish-versatile PCR primers [21-27] and 18 taxon-specific primers (Additional file 2) were used in various combinations to amplify contiguous, overlapping segments of the entire mtDNA for each of the six new cichlid species. The long PCR and subsequent short PCRs were performed as described previously [21,28]. The short-PCR reactions were performed using the GeneAmp PCR System 9700 (Applied Biosystems) and Ex *Taq *DNA polymerase (Takara).

Double-stranded PCR products, treated with ExoSAP-IT (USB) to inactivate remaining primers and dNTPs, were directly used for the cycle sequencing reaction, using dye-labeled terminators (Applied Biosystems) with amplification primers and appropriate internal primers. Labeled fragments were analyzed on Model 3100 and Model 377 DNA sequencers (Applied Biosystems).

### Sequence manipulation

The DNA sequences obtained were edited and analyzed using EditView 1.0.1, AutoAssembler 2.1 (Applied Biosystems) and DNASIS 3.2 (Hitachi Software Engineering Co. Ltd.). Individual gene sequences were identified and aligned with their counterparts in 48 previously published mitogenomes. Amino acid sequences were used to align protein-coding genes, and standard secondary structure models for vertebrate mitochondrial tRNAs [29] were consulted for the alignment of tRNA genes. The 12S and 16S rRNA sequences were initially aligned using clustalX v. 1.83 [30] with default gap penalties and subsequently adjusted by eye using MacClade 4.08 [31].

The ND6 gene was excluded from the phylogenetic analyses because of its heterogeneous base composition and consistently poor phylogenetic performance [22]. The control region was also excluded because positional homology was not confidently established among such distantly-related species. The third codon positions of protein genes were excluded because of their extremely accelerated rates of change that may cause high levels of homoplasy. After the exclusion of unalignable parts in the loop regions of tRNA genes, as well as the 5' and/or 3' end regions of protein genes, all gene sequences were concatenated to produce 10,034-bp sites (6962, 1402, and 1670 positions for protein-coding, tRNA, and rRNA genes, respectively) for phylogenetic analyses.

### Phylogenetic analyses

Phylogenetic trees were reconstructed using partitioned Bayesian and maximum likelihood analyses. Partitioned Bayesian phylogenetic analyses were performed using MrBayes 3.1.2 [32]. We set four partitions (first codon, second codon, tRNA, and rRNA positions). The general time-reversible model, with some sites assumed to be invariable and variable sites assumed to follow a discrete gamma distribution (GTR + I + Γ; [33]), was selected as the best-fit model of nucleotide substitution by MrModeltest 2.2 http://www.abc.se/~nylander/[34]. The Markov chain Monte Carlo (MCMC) process was set so that four chains (three heated and one cold) ran simultaneously. We ran the program for 3,000,000 metropolis-coupled MCMC generations on each analysis, with tree sampling every 100 generations and burn-in after 10,000 trees.

Partitioned maximum likelihood (ML) analyses were performed with RAxML ver. 7.0.3 [35], a program implementing a novel, rapid-hill-climbing algorithm. For each dataset, a rapid bootstrap analysis and search for the best-scoring ML tree were conducted in one single program run, with the GTR + I + Γ nucleotide substitution model. The rapid bootstrap analyses were conducted with 1000 replications, with four threads running in parallel.

Statistical evaluation of alternative phylogenetic hypotheses was done using TREE- PUZZLE 5.2 [36], using the two-sided Kishino and Hasegawa (KH) [37] test, the Shimodaira and Hasegawa (SH) [38] test, and Bayes factors [39,40]. We used the GTR + I + Γ model and its parameters optimized by MrModeltest 2.2.

### Divergence time estimation

For the divergence time estimation, *multidistribute *program [41] was used by assuming a topological relationship thus obtained, but without assuming the molecular clock (i.e., by allowing heterogeneity in molecular evolutionary rate along branches). Upper and/or lower time constraints at selected nodes were set for the Bayesian MCMC processes to estimate divergence times (including means and 95% credibility ranges) and relative rates at ingroup nodes. We set the partitioning as described above and first used PAML [42] to optimize the parameters of model F84 and the gamma distribution for eight categories to account for site heterogeneity. *Estbranches *and *multidivtime *programs were then used to estimate divergence times. We used 21 fossil-based time constraints assignable to diverse teleostean lineages (Table 2).

**Table 2:** Maximum (U) and minimum (L) time constrains (MYA) used for dating at nodes in Fig. 2

Node	Constraint	Reference information
A	L416	*Psarolepis *fossil (the earliest Sarcopterygii) from Ludlow (Silurian) [2]*Lophosteus *and *Andreolepis *fossils (the earliest Actinopterygii) from Ludlow (Silurian) [64]
A	U528	Probable divergence time between chondrichthyans and osteichthyans (528 MYA), based on both fossils and molecules [58]
B	L392	Stem-actinopterans known from the Givetian/Eifelian boundary [57]
B	U450	Probable divergence time between sarcopterygians and actinopterygians (450 MYA), based on both fossils and molecules [6,58]
C	L345	Tournasian *Cosmoptychius *as the earliest stem-group neopterygian [57]
C	U392	Estimated divergence time between polypterids and actinopterans [57]
D	L130	*Protosephuru *(paddlefish) from Hauterivian (Cretaceous) [57]
E	L284	*Brachydegma *from early Permian [57]
F	L136	Stem-hiodontid *Yambiania *from the Lower Cretaceous [57]
G	L112	Osteoglossoid fossil from the Aptian (Cretaceous) [4]
H	L151	Stem-elopomorph *Elopsomolos *from the Kimmeridgian (Jurassic) [57]
I	L90	Albuloid fossil from the Cenomanian (Cretaceous) [4]
J	L50	Anguillid and congrid fossils from the Ypresian (Tertiary) [4]
K	L146	Stem-ostariophysan *Tischlingerichthys *from Tithonian (Jurassic) [57]
L	L57	Clupeid fossil from the Thanetian (Tertiary) [4]
M	L50	Cyprinid fossil from the Ypresian (Tertiary) [4]
N	L74	Esociform fossil from the Campanian (Cretaceous) [4]
O	L94	Polymixiid fossil from the Cenomanian (Cretaceous) [4]
P	L50	Pleuronectiform fossil from the Ypresian (Tertiary) [4]
Q	L98	Tetraodontiform fossil from the Cenomanian (Cretaceous) [2]
R	L32	Estimated divergence time between *Takifugu *and *Tetraodon *[2]

## Results and discussion

### Mitochondrial genomes of cichlids

We determined complete or nearly complete mtDNA nucleotide sequences for six new cichlids from Africa, South America, Madagascar, and Indo/Sri Lanka (Table 1). The sizes of these genomes ranged from 16,457 to 16,556 bp, including approximately 800 bp in the control region. *Tylochromis polylepis *alone appears to have a somewhat longer control region (approximately 1200 bp) although the exact sequence of the region was unable to be determined because of the long poly-T sequences within the region. We also analyzed the previously published mitogenomic sequences of four cichlid species (Table 1). *Oreochromis mossambicus *(accession no. AY597335) was not included because a congeneric taxon (*Oreochromis *sp.) sequenced by Mabuchi et al. [43] had already been sampled.

All 37 genes encoding two rRNAs, 22 tRNAs, and 13 proteins were identified in these 10 cichlid mitogenomes, basically in the same order and orientation found for most other vertebrates. Transfer RNA genes could be folded into secondary structures typical of vertebrate mitochondrial tRNA [29]. The base composition of cichlid mitogenomes was skewed (data not shown) similarly to those of other vertebrates [44].

### Phylogenetic relationships

**Figure 1:**
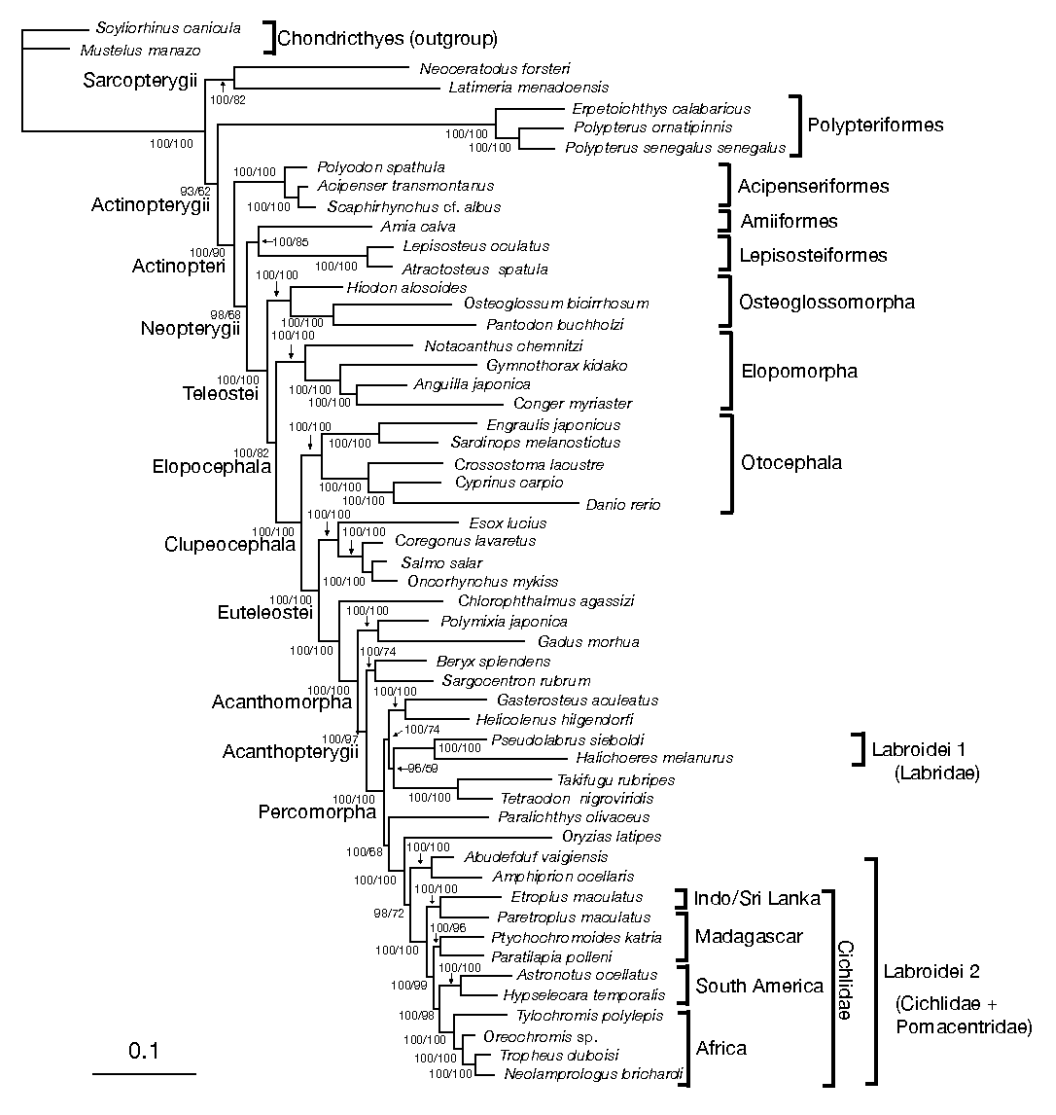
**A Bayesian tree based on mitogenomic DNA sequences**. This is a 50% majority rule consensus tree among 10,000 pooled trees from two independent Bayesian MCMC runs. The data set comprises aligned gap-free nucleotide sequences of 10,034-bp length from 54 taxa, which included 4,887 variable sites and 3,936 parsimony-informative sites. Partitioned Bayesian analyses were conducted using the GTR + I + Γ model and with all model parameters variable and unlinked across partitions. The numerals at internal nodes or branches indicate Bayesian posterior probabilities (left) and maximum likelihood bootstrap probability values (right) from 1000 replicates, respectively (shown as percentage for values above 50%).

Figure 1 shows the phylogenetic relationships inferred from the Bayesian analysis among the 52 bony fishes, estimated with two sharks as an outgroup. The tree topology was identical to that obtained by the partitioned ML analysis (data not shown). These bony fish taxa included two sarcopterygians (coelacanth and lungfish), nine basal actinopterygians (polypterids, acipenseriforms, lepisosteids, and amiid), and 41 teleosts, including 10 cichlids. The phylogenetic relationships obtained for non-cichlid taxa (Fig. 1) were largely consistent with those from previous mitogenomic studies [28,43,45], except for a difference in the sister group of holosteans (lepisosteids and amiid).

Although Inoue et al. [28] suggested that the "Ancient Fish Clade" unites acipenserids, lepisosteids, and amiid, our phylogenetic analysis supports the neopterygian clade (lepisosteids + amiid + teleosts), in agreement with an analysis of nuclear DNA sequences [46] and morphological characters [47]. Relationships between the basal actinopterygians and teleosts were not stable against changes in taxonomic representations and the genes used and varied between the two hypotheses (data not shown). We tentatively assumed the neopterygian relationship for subsequent analyses because this was consistent in both morphological and molecular (based on mitochondrial and nuclear sequences) analyses. However, we also conducted analyses to evaluate how our major conclusions in dating depend on the two alternative phylogenetic relationships (Table 3).

**Table 3:** Comparison of divergence time estimates between different time constraints and studies

Divergence	This study^1^	This study^2^	This study^3^	Yamanoue et al. [55]	Inoue et al. [54]
Cichlidae vs. Pomacentridae	127 (107 – 149)	144 (134 – 154)	137 (115 – 160)	-	-
*Takifugu *vs. *Tetraodon*	70 (55 – 86)	78 (65 – 93)	76 (60 – 94)	73 (57 – 94)	-
Tetraodontidae vs. *Gasterosteus*	154 (131 – 177)	170 (156 – 185)	161 (137 – 185)	192 (153 – 235)	-
Cichlidae vs. *Oryzias*	136 (115 – 159)	152 (141 – 165)	148 (125 – 171)	-	-
Cichlidae/*Oryzias *vs. Tetraodontidae	159 (136 – 183)	176 (163 – 191)	166 (142 – 191)	184 (154 – 221)	-
Percomorpha vs. Beryciformes	182 (157 – 206)	198 (183 – 215)	188 (162 – 214)	206 (174 – 245)	-
Acanthopterygii vs. Gadiformes	191 (166 – 216)	207 (190 – 224)	202 (176 – 229)	223 (191 – 264)	-
Acanthomorpha vs. Protacanthopterygii	249 (223 – 274)	262 (243 – 281)	270 (243 – 294)	280 (240 – 326)	232 (197 – 267)
*Cyprinus *vs. *Danio*	139 (111 – 169)	147 (120 – 174)	135 (107 – 164)	167 (131 – 208)	-
Euteleostei vs. Otocephala	276 (250 – 301)	288 (268 – 307)	291 (264 – 314)	315 (270 – 363)	278 (241 – 314)
Teleostei vs. Amiiformes	360 (339 – 376)	365 (348 – 378)	381 (363 – 392)	390 (340 – 442)	376 (337 – 413)
Sarcopterygii vs. Actinopterygii	428 (417 – 448)	429 (417 – 449)	428 (417 – 449)	470 (415 – 524)	451 (413 – 495)

The means and 95% credibility ranges (in parentheses) are shown for estimated divergence times.

**Figure 2:**
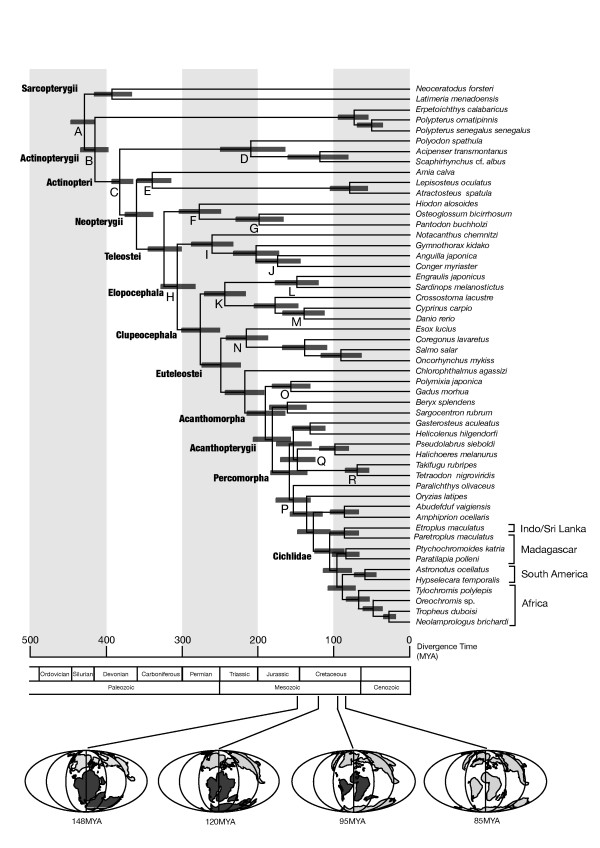
**Divergence times estimated from the partitioned Bayesian analysis**. A posterior distribution of divergence times with 95% credibility intervals (shaded rectangles) was obtained using mitogenomic DNA sequences (10,034 sites). Two sharks (*Scyliorhinus canicula *and *Mustelus manazo*) were used as an outgroup (not shown). The multidistribute program [41] was used to estimate divergence times assuming the tree topology shown in Fig. 1. Letters indicate nodes at which maximum and/or minimum time constraints were set (see Table 2 for details of the individual constraints). Paleogeographical maps at 148 MYA, 120 MYA, 95 MYA, and 85 MYA [50] are shown. Dark-gray areas on the maps represent those being fragmented within Gondwanaland at those times.

^1 ^Without biogeography-based time constraints on cichlid divergences (see Fig. 2).

^2 ^With biogeography-based time constraints on cichlid divergences (see Fig. 4).

^3 ^Without biogeography-based time constraints on cichlid divergences, but assuming the Ancient Fish Clade (see text).

In terms of the relationships among 20 percomorphs containing 14 labroids (two labrids, two pomacentrids, and 10 cichlids), we reconfirmed the polyphyly of Labroidei [43] whereby labrids (designated Labroidei 1 in Fig. 1) and cichlids + pomacentirids (Labroidei 2) appear in separate lineages of teleosts. The non-monophyly of the labroid taxa was supported by a number of nodes with 100% posterior probability and 100% bootstrap values (Fig. 1).

Among the 10 cichlid taxa that we used, four were from Africa, two from South America, three from Madagascar, and one from Indo/Sri Lanka. The tree (Fig. 1) supports the monophyly of Cichlidae and two other continental groups from Africa and South America. Four basal taxa from Madagascar and Indo/Sri Lanka are not monophyletic, and two (*Paretroplus *from Madagascar and *Etroplus *from Indo/Sri Lanka) corresponding to Etroplinae *sensu *Sparks and Smith [16] form a sister group to all other cichlids. The other two Malagasy taxa (*Paratilapia *and *Ptychochromoides*), corresponding to Ptychochrominae *sensu *Sparks and Smith [16], form a sister group to the African + Neotropical clade. These results are consistent with previous molecular studies that used a few mitochondrial or nuclear gene sequences [14-16,48], as well as morphological studies [13].

However, these previous studies did not fully evaluate the statistical significance in rejecting alternative hypotheses of cichlid relationships. We conducted KH and SH tests, as well as a test using Bayes factor. Based on these tests, alternative hypotheses assuming the monophyly of Malagasy + Indo/Sri Lankan cichlids (constraint 1), Old World cichlids (constraint 2), and African + Indo/Sri Lankan cichlids (constraint 3) are all very unlikely (Table 4). These results provide statistical support for the paraphyletic assemblage of the Malagasy + Indo/Sri Lankan taxa to the African + Neotropical clade.

**Table 4:** Test of alternative phylogenetic hypotheses for continental cichlid groups

Topological constraint	pKH	pSH	2 ln Bayes factor
Best as in Fig. 1	1.000	1.000	
Constraint 1: monophyly of Madagascar and Indo/Sri Lanka (Tree 1)	0.006**	0.043*	65.4*
Constraint 2: monophyly of Africa, Madagascar and Indo/Sri Lanka (Tree 2)	0.001**	0.002**	125.1*
Constraint 3: monophyly of Africa and Indo/Sri Lanka (Tree 3)	0.000**	0.000**	297.2*

Probabilities for constrained trees were assessed using the Kishino-Hasegawa (pKH) and Shimodaira-Hasegawa (pSH) tests and the Bayes factor. Single asterisks indicate significant rejection (p < 0.05) and double asterisks indicate highly significant rejection (p < 0.01) of the corresponding hypothesis. We used the traditional criterion of 2 ln Bayes factor over 10 (with an asterisk), indicating very strong evidence against an alternative hypothesis [39]. Constrained trees are the following: Tree 1: ((((*Oreochromis *sp., (*Tropheus duboisi*, *Neolamprologus brichardi*)), *Tylochromis polylepis*),(*Astronotus ocellatus*, *Hypselecara temporalis*)),((*Etroplus maculatus*, *Paretroplus maculatus*),(*Ptychochromoides katria*, *Paratilapia polleni*))); Tree 2: ((((*Oreochromis *sp.,(*Tropheus duboisi*, *Neolamprologus brichardi*)), *Tylochromis polylepis*),((*Etroplus maculatus*, *Paretroplus maculatus*),(*Ptychochromoides katria*, *Paratilapia polleni*))),(*Astronotus ocellatus*, *Hypselecara temporalis*)); and Tree 3: (((((*Oreochromis *sp.,(*Tropheus duboisi*, *Neolamprologus brichardi*)), *Tylochromis polylepis*), *Etroplus maculatus*),((*Astronotus ocellatus*, *Hypselecara temporalis*),(*Ptychochromoides katria*, *Paratilapia polleni*))), *Paretroplus maculatus*).

If Cichlidae originated in Cenozoic Africa and migrated into South America, Madagascar, and India via saltwater dispersal [19,49], Malagasy/Indo Sri Lankan and/or Neotropical taxa would probably be nested in the African clade, and alternative relationships (e.g., those corresponding to constraints 2 and 3) would likely appear. However, these relationships were not found, thus supporting the vicariant divergence scenario [13,14,18], at least from a topological standpoint.

### Timing of cichlid divergences

We conducted divergence time estimation among 54 bony fishes, including 10 cichlids (Fig. 2). Twenty-one time constraints based on extensive fossil evidence for bony fishes (Table 2) were used. Following the advice of Benton and Donoghue [2] to set fossil-based time constraints as hard lower boundaries and soft upper boundaries, we chose older values for upper boundaries. We estimated the divergence between African + Neotropical cichlids and Malagasy + Indo/Sri Lankan (ptychochrominae) cichlids to be approximately 96 MYA (78–115 MYA at 95% credibility). The divergences of African vs. Neotropical cichlids and Malagasy vs. Indo/Sri Lankan cichlids within the Etroplinae were estimated to be approximately 89 MYA (72–108 MYA) and 87 MYA (69–106 MYA), respectively.

We then compared the estimated divergence times among cichlids and the probable times of continental fragmentation based on geological evidence. The divergence time between Malagasy and Indo/Sri Lankan taxa within Etroplinae (~87 MYA: 69–106 MYA) is very close to the time of separation between Madagascar and India (85–95 MYA) [50,51]. The divergence time estimated between African and Neotropical clades (~89 MYA: 72–108 MYA) is also close to the time of separation between African and South American landmasses (~100 MYA) [50,51]. The divergence time between African + Neotropical cichlids and Malagasy ptychochrominae cichlids (~96 MYA: 78–115 MYA) appears to be somewhat more recent than the time generally accepted for the complete separation of the Indo-Madagascar landmass from Gondwanaland (120–130 MYA) [50,51]. However, some studies [52] have postulated an extended connection between India and Antarctica by approximately 112 MYA, which is within the 95% credibility range for the African/Neotropical vs. ptychochrominae cichlid divergence. Taken together, these results are consistent with the vicariant divergence of continental cichlid groups during Cretaceous times and argue against their Cenozoic dispersal.

Vences et al. [19] calibrated a molecular clock for cichlids that assumed that the divergence time of the most basal endemic lineages in East African Rift lakes (e.g., Tanganyika) corresponds to the geological estimate of the age of the lakes. These estimated divergence times between continental cichlid clades were all in the Cenozoic (rather than the Mesozoic, as we demonstrate in Fig. 2) and supported the hypothesis of long-distance Cenozoic transmarine dispersal of cichlids. This view of the Cenozoic (or latest Cretaceous) origin and transmarine dispersal of cichlids has also been supported by some biogeographers [49] because it is consistent with cichlid fossil records, which first occur in South America and Africa in the Eocene [20,53]. However, the clock-based dating procedures of Vences et al. [19] present some problems. The strict molecular clock may not hold for all cichlid lineages [15], and the premise that the oldest endemic cichlid divergence is synchronized with the formation of the lakes may not be valid. Some lineages that had diverged outside the lake may have immigrated in parallel [7]. In addition, there is no definitive, geologically based time estimate for the formation of the lakes.

More recently, Genner et al. [7] used two mitochondrial (cytochrome *b *and 16S rRNA) and one nuclear (TMO-4C4) gene fragments to estimate the divergence times among cichlids. When the cichlid divergence by Gondwanan vicariance was assumed, the resultant divergence times were more consistent with those estimated with time constraints from previous paleontological and molecular studies [2,54-57] than when the Cenozoic cichlid divergence was assumed based on fossil records.

Although we concur on the Gondwanan origin and vicariant divergence of cichlids, Genner et al. [7] evaluated this biogeographic hypothesis somewhat indirectly, in that the fitness of estimated times of cichlid divergences to those obtained with time constraints from previous studies was qualitatively compared between alternative assumptions on cichlid biogeography. We evaluated cichlid divergence times more directly by using longer mitogenomic sequence data and dozens of non-cichlid taxa, allowing us to set many time constraints purely from the paleontological data and providing additional evidence for an ancient cichlid divergence on Gondwanaland, despite the general paucity of the Mesozoic and Cenozoic paleontological record on bony fishes.

### Gondwana fragmentation as time constraints

**Figure 3:**
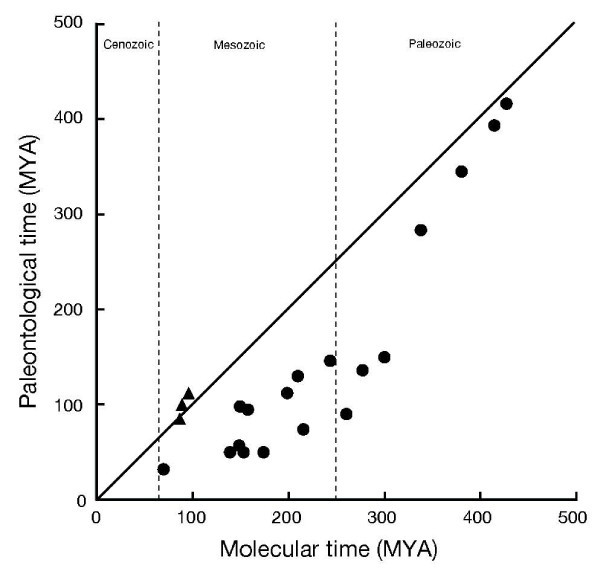
**Comparison of paleontological and molecular estimates of divergence times**. Minimum estimates of divergence times deducible from fossil records (see Table 2) were plotted as closed circles against molecularly estimated divergence times (mean values for the divergence times shown in Fig. 2). Closed triangles show plots of the timing of continental breakups against the molecular time estimates of cichlid divergences between the corresponding continents (data taken from Fig. 2). The timings used for complete continental breakups are 112 MYA for (Africa + South America) vs. (Madagascar + Indo/Sri Lanka), 100 MYA for Africa vs. South America, and 85 MYA for Madagascar vs. Indo/Sri Lanka [50-52]. The solid line indicates a 1:1 relationship between paleontological and molecular time estimates.

In Figure 3, minimum time constraints based on fossil records (see Table 2) are plotted against molecular time estimates of the corresponding divergences (values taken from Fig. 2). In this figure, minimum age estimates of Gondwanan fragmentations are also plotted against the corresponding molecular time estimates of continental cichlid groups. It should be noted here that the latter data points reflecting Gondwanan fragmentation history (closed triangles) are plotted well on the line of 1:1 relationship whereas most of the data points reflecting fossil records (closed circles) are considerably below the line of the 1:1 relationship. This pattern suggests that Gondwana fragmentation history that is congruent with the cichlid phylogeny can be effective time constraints better than most of the Mesozoic and Cenozoic fossil records used here.

Among the fossil data points, four data points in the Paleozoic show a fairly good 1:1 relationship, whereas other points mostly in the Mesozoic are considerably below the line of 1:1 relationship. This might mean that the Mesozoic fossils do not really represent the oldest fossil for the corresponding lineages whereas this is not the case for older Paleozoic lineages. This situation is somewhat reminiscent of the apparent relative paucity of Mesozoic fossil evidence of tetrapods (mammals and birds) [58].

Several papers have noticed that molecular time estimations are consistently older than paleontological ones [2,3,5-7,59]. Benton and Ayala [60] have pointed out four pervasive biases that make molecular dates too old: i) too old calibration dates based on previous molecular studies; ii) undetected fast-evolving genes; iii) ancestral polymorphism that is maintained through long evolutionary period; and iv) asymmetric distributions of estimated times, with a constrained younger end but an unconstrained older end (this is caused because rates of evolution are constrained to be nonnegative, but the rates are unbounded above zero).

The first factor is not the case for the present study, because we did not use the calibration dates based on previous molecular studies, but used only those based on fossil records. The third factor would be the case when the used genomic regions are under the long-term balancing selection, but no mitochondrial gene has been reported to be under such selection. Regarding the second and fourth factors, we believe that they are also not the case for this study, because we used mitogenomic sequence data excluding peculiarly rapid evolving region (e.g., the control region), and because each mitochondrial gene used here was tested to perform well for dating vertebrate (tetrapod) divergences [61]. According to Benton and Ayala [60], for reliable dating "careful choice of genes may be a more appropriate strategy (than the larger data strategy), with a focus on long and fast-evolving (yet alignable) sequences." Our present study based on nearly whole mitogenomic sequence data fairly accommodates such condition.

### Improved dating of teleostean divergences

**Figure 4:**
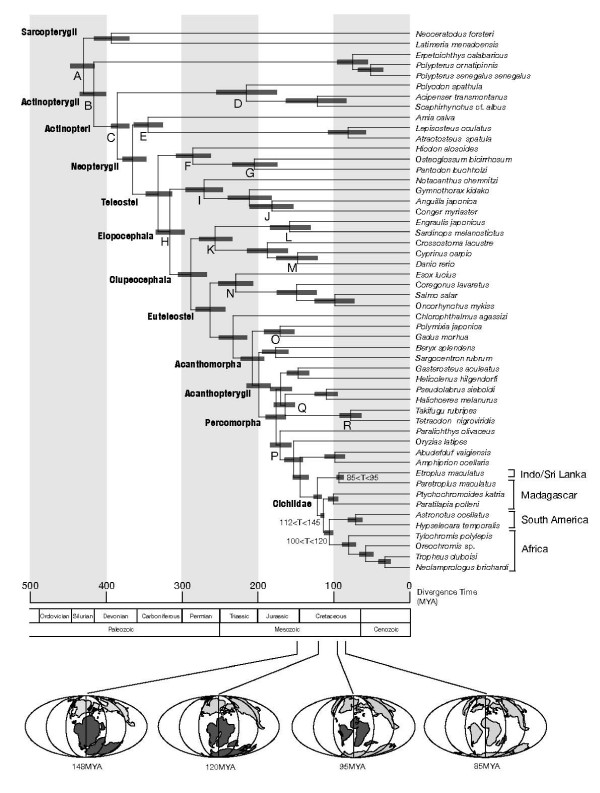
**Divergence times estimated from the partitioned Bayesian analysis using both paleontological time constraints (Table 2) and biogeographical assumptions for the divergences of continental cichlid groups**. The added time constraints on cichlid divergences are as follows: 112 MYA (lower) and 145 MYA (upper) for (Africa + South America) vs. (Madagascar + Indo/Sri Lanka); 100 MYA (lower) and 120 MYA (upper) for Africa vs. South America; and 85 MYA (lower) and 95 MYA (upper) for Madagascar vs. Indo/Sri Lanka [50-52]. See Fig. 2 legend for other details.

We then conducted the divergence time estimation using the Gondwanan vicariance assumption regarding cichlids as additional time constraints (Fig. 4). Compared to the results shown in Figure 2 (without the additional time constraints), the means of estimated divergence times at various nodes are similar or somewhat larger (= 18 million years; see Table 3). However, the 95% credibility ranges of the estimated times overlap well between the two results, and the differences in mean values are not large, compared to potential error ranges in other elements, such as stochastic errors in molecular evolution and errors in dating fossils.

The addition of the cichlid constraints appears to shorten the 95% credibility intervals of the time estimates, especially for divergences occurring within Acanthomorpha 100–200 MYA. For example, our Figure 2 and Yamanoue et al. [55] estimated the divergence time of torafugu (Tetraodontiformes) and medaka (Beloniformes) to be approximately 159 (136–183) MYA and 184 (154–221) MYA, respectively. The cichlid constraints considerably narrowed the 95% credibility interval to 176 (163–191) MYA (Table 3), and also increased the precision of time estimates for other nodes. The use of ample molecular data from mitogenomic sequences also helped to narrow the credibility interval. For example, Kumazawa et al. [5] used two mitochondrial genes (NADH dehydrogenase subunit 2 and cytochrome *b*) and estimated the divergence between torafugu and zebrafish at 284 ± 28 (mean ± standard deviation) MYA, whereas our whole mitogenomic data set showed the divergence at 288 (268–307) MYA (Table 3).

## Conclusion

We estimated the divergence times of major cichlid lineages as part of the longer evolutionary history of teleostean fishes. Our results and those of a recent molecular study based on both mitochondrial and nuclear data sets [7] support a vicariant history of cichlid divergences, while other researchers [19] have argued for the dispersal hypothesis. We presented additional strong evidence for the vicariant hypothesis and propose that the vicariant assumption can be used to generate time constraints to date other teleostean divergences in both deeper (100–300 MYA) and shallower (< 100 MYA) time ranges.

This could be a significant contribution toward the reliable dating of teleostean divergence times in light of the scarcity of teleostean fossil records in the Mesozoic and later (see above) and the probable deviation of molecular evolutionary rates of fishes from those of tetrapods [5,62], for which molecular evolutionary rates are more reliably studied using ampler fossil records. A further exploration of biogeography-based time constraints for other groups of freshwater fishes that could be reasonably incorporated into the dating study (e.g. rainbowfishes [63]) would be expected to increase the accuracy and precision of teleostean divergence time estimates.

## Authors' contributions

YK, MM, and MN designed the study. YA carried out the molecular work and analyzed the data. MM and KM participated in the data analysis. YA and YK drafted the original manuscript. MM and KM contributed to the improvement of all versions of the manuscript. The publication fee was provided by YK. All authors read and approved the final manuscript.

## Supplementary Material

Additional File 1 **List of species used, with database accession numbers**. Classifications follow Nelson [11]. Click here for file

Additional File 2 **Cichlid-specific primers for PCR and sequencing**. H and L indicate the orientation of the primers. The locations of the primers are shown with the names of the targeted genes. Click here for file
