# Supplementary material for: Mitogenomic evaluation of the historical biogeography of cichlids toward reliable dating of teleostean divergences
Source: BMC Evol Biol. 2008 Jul 23;8:215. doi: 10.1186/1471-2148-8-215 (PMC2496912; doi:10.1186/1471-2148-8-215)
Supplement: Additional File 1 — List of species used, with database accession numbers. Classifications follow Nelson [11]. [file 1471-2148-8-215-S1.doc]

**Additional file 1 - List of species used, with database accession numbers**

Order Family Species Accession No.

Outgroups

Carcharhiniformes Scyliorhinidae *Scyliorhinus canicula* Y16067

Triakidae *Mustelus manazo* AB015962

Coelacanthiformes Latimeriidae *Latimeria menadoensis* AP006858

Ceratodontiformes Ceratodontidae *Neoceratodus forsteri* AJ584642

Polypteriformes Polypteridae *Polypterus ornatipinnis* AP004351

*Polypterus senegalus senegalus* AP004352

*Erpetoichthys calabaricus* AP004350

Acipenseriformes Acipenseridae *Acipenser transmontanus* AB042837

*Scaphirhynchus* cf. *albus* AP004354

Polyodontidae *Polyodon spathula* AP004353

Lepisosteiformes Lepisosteidae *Lepisosteus oculatus* AB042861

*Atractosteus spatula* AP004355

Amiiformes Amiidae *Amia calva* AB042952

Hiodontiformes Hiodontidae *Hiodon alosoides* AP004356

Osteoglossiformes Osteoglossidae *Osteoglossum bicirrhosum* AB043025

*Pantodon buchholzi* AB043068

Albuliformes Notacanthidae *Notacanthus chemnitzi* AP002975

Anguilliformes Anguillidae *Anguilla japonica* AB038556

Muraenidae *Gymnothorax kidako* AP002976

Congridae *Conger myriaster* AB038381

Clupeiformes Engraulidae *Engraulis japonicus* AB040676

Clupeidae *Sardinops melanostictus* AB032554

Cypriniformes Cyprinidae *Cyprinus carpio* X61010

*Danio rerio* AC024175

Balitoridae *Crossostoma lacustre* M91245

Salmoniformes Salmonidae *Coregonus lavaretus* AB034824

*Salmo salar* U12143

*Oncorhynchus mykiss* L29771

Esociformes Esocidae *Esox lucius* AP004103

Aulopiformes Chlorophthalmidae *Chlorophthalmus agassizi* AP002918

Polymixiiformes Polymixiidae *Polymixia japonica* AB034826

Gadiformes Gadidae *Gadus morhua* X99772

Beloniformes Adrianichthyidae *Oryzias latipes* AP004421

Beryciformes Berycidae *Beryx splendens* AP002939

Holocentridae *Sargocentron rubrum* AP004432

Gasterosteiformes Gasterosteidae *Gasterosteus aculeatus* AP002944

Scorpaeniformes Scorpaenidae *Helicolenus hilgendorfi* AP002948

Perciformes Cichlidae *Oreochromis* sp. AP009126

*Neolamprologus brichardi* AP006014

*Tropheus duboisi* AP006015

*Astronotus ocellatus* AP009127

*Paretroplus maculatus* AP009504

*Etroplus maculatus* AP009505

*Hypselecara temporalis* AP009506

*Ptychochromoides katria* AP009507

*Paratilapia polleni* AP009508

*Tylochromis polylepis* AP009509

Pomacentridae *Abudefduf vaigiensis* AP006016

*Amphiprion ocellaris* AP006017

Labridae *Pseudolabrus sieboldi* AP006019

*Halichoeres melanurus* AP006018

Pleuronectiformes Paralichthyidae *Paralichthys olivaceus* AB028664

Tetraodontiformes Tetraodontidae *Takifugu rubripes* AJ421455

*Tetraodon nigroviridis* AP006046

Classifications follow Nelson [11].
