# Supplementary material for: Mitogenomic evaluation of the historical biogeography of cichlids toward reliable dating of teleostean divergences
Source: BMC Evol Biol. 2008 Jul 23;8:215. doi: 10.1186/1471-2148-8-215 (PMC2496912; doi:10.1186/1471-2148-8-215)
Supplement: Additional File 2 — Cichlid-specific primers for PCR and sequencing. H and L indicate the orientation of the primers. The locations of the primers are shown with the names of the targeted genes. [file 1471-2148-8-215-S2.doc]

**Additional file 2 - Cichlid-specific primers for PCR and sequencing**

Name Sequence (5´ to 3´)

H-12s-Cichlid CCTTCCGGTACACTTACCAT

H-ND2-Cichlid TGKGTTTGGTTTARSCCKCCTCAKCC

H-CO1-Cichlid GACRTAGTGRAARTGGGCRAC

H-ATP6-Cichlid AAGCGRTTRATAAACCAYCCTTGRAGKG

H-CO3-Cichlid TGRATTGCTTSTTTTCGRTKMCCYTC

H-ND4-Cichlid CGTATTATTCCGTANCCNCC

H-ND5-1-Cichlid GCTTCAATAATRGCGTCTTTNGA

H-ND5-2-Cichlid TGGGWGGCRATGGYTTGNCC

H-CYB-Cichlid AAKCCKCCTCARATTCATTG

L-12S-Cichlid AAACCCAAAGGACTTGGCGGTG

L-CO1-Cichlid ATGGACGTAGACACACGVGC

L-ATP6-Cichlid ATACGAAAYCARCCYACCCAYGC

L-CO3-Cichlid CACACVCCHCCYGTCCAAAA

L-ND4L-Cichlid GGCCTRGCATTYCAYCGAAC

L-tRNA-His-Cichlid CGHTGAGGHAAYCARACAGAACG

L-ND5-Cichlid TTGCYATAGCMTGAAYYGCAAC

L-ND6-Cichlid TAAACAGCYCGAAGRGCCCC

L-CR-Cichlid CAWGCCGRGCRTTCTTTCCAG

H and L indicate the orientation of the primers. The locations of the primers are shown with the names of the targeted genes.
